# Methods for evaluating cervical range of motion in trauma settings

**DOI:** 10.1186/1757-7241-20-50

**Published:** 2012-08-02

**Authors:** Sarah Voss, Michael Page, Jonathan Benger

**Affiliations:** 1 Faculty of Health and Life Sciences, University of the West of England, Glenside Campus, Blackberry Hill, Bristol, UK, BS16 1DD; 2 Great Western Ambulance Service NHS Trust, Chippenham, UK

**Keywords:** Cervical range of motion, Cervical vertebrae injuries, Immobilisation methods, Evaluation, Assessment, Measurement

## Abstract

Immobilisation of the cervical spine is a common procedure following traumatic injury. This is often precautionary as the actual incidence of spinal injury is low. Nonetheless, stabilisation of the head and neck is an important part of pre-hospital care due to the catastrophic damage that may follow if further unrestricted movement occurs in the presence of an unstable spinal injury. Currently available collars are limited by the potential for inadequate immobilisation and complications caused by pressure on the patient’s skin, restricted airway access and compression of the jugular vein. Alternative approaches to cervical spine immobilisation are being considered, and the investigation of these new methods requires a standardised approach to the evaluation of neck movement. This review summarises the research methods and scientific technology that have been used to assess and measure cervical range of motion, and which are likely to underpin future research in this field. A systematic search of international literature was conducted to evaluate the methodologies used to assess the extremes of movement that can be achieved in six domains. 34 papers were included in the review. These studies used a range of methodologies, but study quality was generally low. Laboratory investigations and biomechanical studies have gradually given way to methods that more accurately reflect the real-life situations in which cervical spine immobilisation occurs. Latterly, new approaches using virtual reality and simulation have been developed. Coupled with modern electromagnetic tracking technology this has considerable potential for effective application in future research. However, use of these technologies in real life settings can be problematic and more research is needed.

## Introduction

Serious injuries, particularly those resulting from road traffic collisions, are increasing worldwide with more than one million deaths annually [1]. In many countries with developed pre-hospital and in-hospital trauma systems it is usual practice to apply a cervical collar to the neck of patients who have sustained blunt trauma, particularly those with head injury, to reduce neck movement prior to definitive assessment. This practice is supported by widely accepted international trauma guidelines, such as those promoted in the “Advanced Trauma Life Support” (ATLS) course [2]. The theory behind the use of cervical collars is that the trauma patient may have sustained an unstable cervical spine injury, and that the application of a collar reduces the risk of additional neurological damage before the presence or absence of such an injury can be reliably determined, either clinically or radiologically.

However, the proposed benefits of cervical collars are theoretical only, and have never been shown to exist in clinical practice [3], with one study concluding that spinal immobilisation had little or no effect on neurological outcome for blunt trauma injury patients [4]. On the contrary, spinal immobilisation following penetrating trauma has been associated with increased mortality [5]. Furthermore, cervical collars are known to cause a number of complications including respiratory compromise, pressure necrosis and raised intracranial pressure [3]. As new approaches to potential cervical spine injury are introduced, along with new immobilisation devices, it is important to consider ways in which these can be usefully assessed and compared, particularly in relation to their ability to limit cervical movement. Furthermore, with the application of improved technology to the measurement of neck movement it becomes possible to address a number of related questions. Shafer and Naunheim [6] recently reported a preliminary study in which a six-camera motion capture system was used to examine cervical movement during mock extrications from a vehicle, suggesting that for conscious patients neck movement may be minimised by applying a cervical collar and inviting the patient to remove themselves from the car, rather than employing a more complex and time-consuming extrication technique.

Further research is therefore needed both to evaluate the effectiveness of existing and new immobilisation devices, and also to investigate alternative approaches to cervical spine management in trauma patients. In order to carry out this research, there is a need to identify and employ standardised methods of assessing and measuring cervical range of motion. This is the subject of the following review.

### Aim

This review aims to provide a brief overview of relatively recent research in the evaluation of cervical spine range of motion (CROM), with particular reference to the testing of neck immobilisation devices. It is divided into two main sections:

1) Research methods that have been used to assess CROM, and

2) Scientific methods that have been used to measure and record CROM.

## Methods

### Data sources and search strategy

A systematic search of international literature was conducted in AMED, Cochrane Library, CINAHL Plus, EMBASE, MEDLINE, PubMed, and SPORTDiscuss. Boolean/Phase mode was applied using the terms cervical motion OR cervical range of motion OR cervical movement OR cervical range of movement AND evaluat* OR compar* OR assess*.

### Inclusion and exclusion criteria

Due to a lack of high quality research in the area all study types were included. Peer reviewed articles published in English between January 2001 and January 2012 were included in the search. Articles were excluded if the primary focus of the evaluation was to assess neck pain, chronic disorders or range of motion in children. Studies were included only if they were considered relevant to the study objectives. A summary of papers included in the study is available in Additional file 1: Appendix. Information was extracted from each study on: methods used to assess CROM (11 papers included) [6-16]; technologies used to evaluate and quantify CROM (29 papers included) [7-9,11,13,16-39]; the statistics supporting the main findings of the study.

## Results

**Figure 1:**
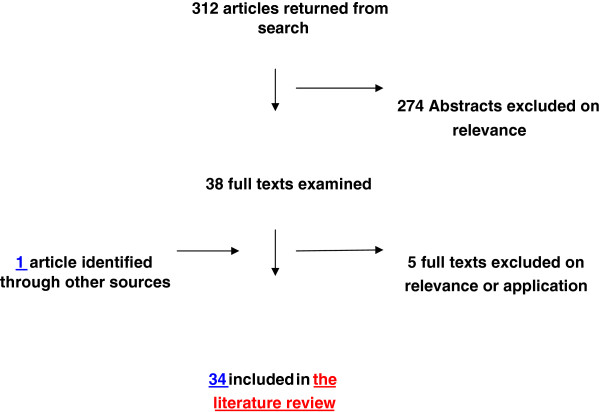
Flow diagram summarising the results of the literature search.

A flow diagram summarising the results of the literature search is shown in Figure 1

### CROM domains

CROM is generally described in the following terms:

Extension (E)

Flexion (F)

Right rotation (RR)

Left Rotation (LR)

Left lateral flexion (LLF)

Right lateral flexion (RLF)

#### Research methods to assess CROM

There has been much research to assess CROM; this has been conducted to test both the efficacy of different devices and also to compare CROM during different movement procedures.

When comparing devices, most authors compare CROM in all the domains, before and after application of a cervical collar [7-9]. Volunteers are instructed to actively move their necks as much as possible in the directions of flexion, extension, rotation, and lateral flexion while lying supine or seated.

Hoppenbrouwers et al [10] used both passive and active methods to assess CROM using two independent examiners. For the passive tests, the subject was instructed to allow the tester to move the subject's neck through the motions without resisting. The authors do not specify the amount of force applied for the passive testing, but each movement was carried out twice by each examiner. For the active tests, the subject was instructed to rotate his or her head as far as possible in the direction of the motion being tested. The authors conclude that reproducibility for active and passive range of motion is good for extension but poor for lateral flexion.

Numerous investigations, similar to those described above, have attempted to assess the general ability of cervical collars to restrict or reduce both segmental and overall spinal motion. However, these studies were designed to evaluate the ability of collars to prevent the cervical spine from moving (be it actively or passively) through large ranges of extreme motion.

Del Rossi et al [11] suggest that the results from such studies cannot be used to establish the relative usefulness of cervical collars:

“Normally, the purpose of an extrication-type collar is to help minimize or control the (presumably) small amount of spinal motion that may be unintentionally generated during the pre-hospital stages of emergency care. Extrication collars are thus more aptly studied if the experimental protocol that is developed includes an external loading condition that results in the production of spinal motion that approximates the quality and quantity of motion generated in real-life situations.” (Pg. 620).

Earlier research by Richter et al [12] addressed this issue by using a 50 N load to test the ability of four collars to control the amount of motion produced between unstable cervical spine segments. The authors surgically created an experimental lesion at C1-2 and C2-3 in four cadavers. However, it would not be practical to apply this methodology in vivo; the authors recognise that factors such as patient comfort and compliance are beyond the limits of a biomechanical study.

Del Rossi et al [11] also used cadavers of differing morphologies with surgically created spinal lesions (complete segmental injury resulting in complete instability) to compare 3 collars. They recruited 6 participants to execute log rolls and lift-and-slide transfer techniques and assessed CROM using electromagnetic tracking. Their findings indicated that there were no significant differences in the 3 collars in limiting CROM during either procedure (the three devices had similar characteristics). Moreover, there were no significant differences between any of the collar treatments and the control condition (no collar). The authors acknowledge a number of limitations in their methodology. These include issues with the use of cadavers; muscle changes resulting in dissimilarity in motion to living patients and the surgical creation of one type of injury reducing the generalisability of findings. They recommend that replication of research of this nature should use a larger sample size and cervical devices with different characteristics. The latter recommendation is derived from their observation that significantly greater CROM was generated by log roll than the lift-and-slide procedure.

Sarig-Bahat et al [13] also acknowledge that conventional methods for assessing CROM in vivo are limited by the subject’s response to an assessor’s instructions. They argue that in day-to-day life, head movement is generally an involuntary response to multiple stimuli. Therefore, there is a need for a more functional assessment method, using sensory stimuli to elicit spontaneous neck motion. They suggest that Virtual Reality (VR) attributes may provide a methodology for achieving this goal. They developed a VR based testing protocol for the assessment of CROM and compared this to conventional assessment methods (the authors do not specify if the conventional assessment was passive, active or scripted). They used a computer system that included a head mounted display and participants were required to target a virtual spray at a fly that appeared on the display. Changes in head position (i.e. neck motion) controlled the location of the canister nozzle similar to the way mouse movements control a cursor. The fly appeared at the top, bottom, right and left sides of the screen to stimulate cervical extension (E), flexion (F), right rotation (RR), and left rotation (LR), respectively. CROM was recorded using 3D electromagnetic technology. The authors found that inter- and intra-tester reliability was achieved for both the VR method and the conventional method of assessment, but the VR method was more precise than conventional assessment. One point to note is that LLF and RLF were not assessed. However, this VR method has also been found to be valid and reliable in the clinical assessment of deficits in movement control [14].

The characteristics of different devices will vary according to their purpose; rehabilitation collars require different qualities from trauma collars. There is a need to evaluate the effectiveness of cervical orthoses in the environment in which they will be employed; i.e. outside of the laboratory [15]. James et al [7] completed a study to quantify CROM during the application of four different rigid collars in a simulated, athletic- related, spine-board situation. Similarly, Krell et al [16] examined differences in spinal movement whilst healthy subject were being placed onto a traditional long backboard and a commercially available scoop stretcher.

As outlined in the introduction, Shafer and Naunheim [6] used a mock extrication scenario to compare different methods of extrication. They developed a mock-up vehicle with the roof removed and compared 4 extrication methods, evaluating CROM with a camera motion capture system. They recruited 3 paramedics and one acted as driver and was extricated by the other two, for each of the methods being investigated. The main limitations acknowledged by the authors were: problems with the use of the camera system and placement of markers resulting in a loss of data; use of a mock-up vehicle (no further detail given); use of medically trained personnel as participants (their medical knowledge was considered a drawback). The limitations of the measurement recording in this study were unfortunate, and the authors encourage future studies to consider other methods.

#### Scientific methods for measuring and recording CROM

Measurement of cervical motion provides substantial information for clinicians about the severity of motion limitation as well as being applicable to the evaluation of new immobilization devices and techniques [17]. Consequently, there has been much research to evaluate the reliability and validity of different measurement techniques.

##### Radiography

Radiographs are used to examine segmental motion confined to a single plane; therefore multiple exams are needed to assess the different domains [18,19]. This is ethically problematic due to the associated radiation exposure, and is not appropriate for developmental research with volunteers.

##### Goniometers and inclinometers

Various commercial measurement devices have been used to measure CROM in the clinical setting [20,21]. Hostler et al [9] used a goniometer and tape measure to compare three cervical immobilization devices on healthy volunteers in a laboratory setting.

The Cervical Range of Motion device (CROM) is a validated piece of equipment for measuring cervical spine range of movement [22-25] and has been used in a number of cervical movement studies [8,26,27]. The device consists of a plastic frame mounted over the nose and ears and secured with a strap. Flexion, extension and lateral flexion are recorded by two gravity goniometers. However, the CROM does not measure rotation and can only be used on participants in an upright position as the measurement system relies on gravity. A study by Schneider et al [28] compared cervical range of movement in seven different orthoses. The authors emphasise the importance of effective immobilisation in both supine and upright positions; it is essential to be able to assess this in order to evaluate a cervical collar.

##### Electromagnetic tracking devices

**Figure 2:**
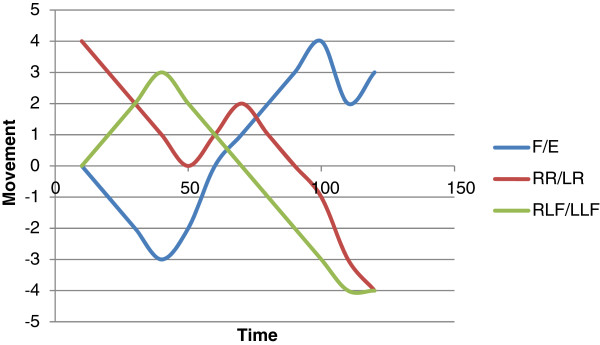
An illustration of how movement in the different domains can be represented with 3D motion analysis (F/E: flexion/extension; RR/LR: right rotation/left rotation; RLF/LLF: right lateral flexion/left lateral flexion).

In recent years there has been increasing interest in more accurate and reliable methods of measuring CROM. This has involved the development and use of non-invasive methods of 3D motion analysis. Sensors are fitted at bony anchor points (e.g. head and sternum) and computer software is used to measure relative movement. These methods produce data to show movements over time in the relative domains. This can be represented graphically, with Figure 2 offering an example, and there are further illustrations in other papers [13]. This form of tracking offers great promise for evaluating CROM when comparing immobilisation devices in both simulated and real conditions. 

Published literature concerned with the evaluation of CROM reports on a variety of electromagnetic systems. The Flock of Birds (Ascension, Burlington, USA) is a sophisticated electromagnetic tracking device, but it requires extensive calibration and is not portable [29,30].

Motion Star (Ascension, Burlington, USA) has been used in research involving simulated scenarios [7,16,31] and the Liberty device (Polhemus Inc., USA) and the Optotrak (Northern Digital, USA) have been used in a variety of cavaderic biomechanical studies [32-36].

An increasingly popular method of electromagnetic tracking is the 3D Fastrak measurement system (Polhemus Inc., USA) [11,13,37]. Rechtine et al [38] used Fastrak to assess movement during hospital bed transfers with different collars. Some recent studies considered Fastrak to be a criterion standard instrument for evaluation of cervical ROM, and have used it for the validation of other methods [23,39].

## Discussion

Assessing CROM with sufficient accuracy to evaluate different neck collars is problematic. In order to compare the efficacy of devices, the physical characteristics of those wearing them (e.g. BMI, height, neck circumference) need to be controlled. There is no commonly accepted methodology for standardising movement, and there is debate about whether subjects should be instructed to move in all directions as far as they can or if the movement should be passive. In terms of active movement, strength and size of the subject as well as interpretation of the instructions given by an assessor are likely to confound the findings. For passive movements there is no accepted degree of force that should be applied, and the reliability of findings to date has been based on the use of a consistent force and assessor for all subjects.

In clinical use, cervical collars need to minimise or control the small amount of spinal motion that may be unintentionally generated during the pre-hospital stages of emergency care. Thus there is a need for an experimental protocol that approximates the quality and quantity of motion generated in real-life situations.

Recent research has focussed on evaluating CROM in more authentic situations using virtual reality or mock extrication. This has the potential to provide a useful functional comparison of different immobilisation devices and techniques, particularly when combined with modern electromagnetic tracking systems which are proving to have both the reliability and accuracy required to usefully compare different approaches to cervical immobilisation. However, a drawback to these technologies is the fact that electromagnetic waves can be distorted by proximity to metal objects [39]. This has the potential to limit their usefulness in both simulated and clinical settings. Thus, there is a need to explore further portable or multiple-modality technology that can be used to accurately evaluate devices and procedures that are designed to protect the cervical spine in trauma care.

## Conclusion

The effective comparison of various devices and techniques to immobilise the cervical spine following trauma requires robust methods to evaluate cervical range of motion in a range of static, dynamic, simulated and clinical situations. None of the current systems are perfect, and controversy remains over the best way to undertake a systematic evaluation. Electromagnetic tracking technology is emerging as the preferred and most reliable approach for laboratory and biomechanical studies. However, electromagnetic measurement may be less useful in simulated and clinical environments as a result of the interference caused by the close proximity of metal. Therefore, the identification of reliable and valid protocols and technologies to measure CROM in these most important settings is a priority for future research. This will in turn facilitate the comparison of different devices and techniques for cervical immobilisation in trauma patients, thereby improving future standards of clinical care.

## Competing interests

The authors declare that they have no competing interests.

## Authors’ contributions

SV conducted the review and drafted the manuscript. MP assisted with the review and inclusion and exclusion of relevant studies. JB helped draft the manuscript and provided clinical context for the aim of this research. All authors read and approved the final manuscript.

## Supplementary Material

Additional file 1: Appendix. Summary of papers included in the review. Click here for file
