# Supplementary material for: Methods for evaluating cervical range of motion in trauma settings
Source: Scand J Trauma Resusc Emerg Med. 2012 Aug 2;20:50. doi: 10.1186/1757-7241-20-50 (PMC3489885; doi:10.1186/1757-7241-20-50)
Supplement: Additional file 1: Appendix. — Summary of papers included in the review. [file 1757-7241-20-50-S1.docx]

Appendix 1: Summary of papers included in the review

|  | **Country** | **Year** | **Ref** | **Design** | **Aim** | **No of primary compar- isons** | **Sample size** | **Measures** | **Comments** |
| --- | --- | --- | --- | --- | --- | --- | --- | --- | --- |
| **Section 1: Research method to assess CROM** | USA | 2009 | 6 | Observational | Extrication technique comparison | 2 | 3 | Camera motion-capture system | Line of sight problems  Limited sample size |
|  | USA | 2004 | 7 | Observational | Collar comparison | 4 | 17 | Active range of motion  Electromagnetic measurement |  |
|  | Netherlands | 2006 | 10 | Test-retest | Comparison of active and passive CROM | 2 | 69 | Examiner judgement | Seated only  Subjective assessment |
|  | Germany | 2001 | 12 | Observational | Device comparison | 4 | 4 | Active range of motion | Applied known force |
|  | Norway | 2010 | 14 | Test-retest  Case control | Assessment evaluation | 2 | 54 | Electromagnetic measurement | Learning effects |
|  | USA | 2005 | 15 | Repeated measures | Spineboard transfer technique | 2 | 13 | Camera motion-capture system | Tested on one casualty |
| **Sections 1 and 2** | USA | 2007 | 8 | Observational | Collar comparison | 4 | 48 | Goniometer | Seated only |
|  | USA | 2009 | 9 | Prospective | Device comparison | 3 | 25 | Gioniometeric  Tape measure | Seated and supine |
|  | USA | 2004 | 11 | Repeated measures | Spineboard transfer technique | 2 | 24 | Electromagnetic measurement | Biomechanical/cavaderic |
|  | Israel | 2009 | 13 | Repeated Measures | Assessment evaluation | 2 | 30 | Electromagnetic measurement | Learning effects  Subject fatigue |
|  | USA | 2006 | 16 | Case control | Spineboard transfer technique comparison | 2 | 31 | Goniometer  Electromagnetic measurement | Instability of sensors |
| **Section 2: Scientific methods for measuring CROM** | Israel | 2008 | 17 | Literature Review | Cervical motion assessment methodology | N/A | 131 | Cervical/head/neck /ROM | PubMed only  Limited search terms |
|  | USA | 2002 | 18 | Case control | Comparison of measurement methods | 3 | 115 | Radiography  Inclinometer  Gioniometer | Single rater |
|  | USA | 2011 | 19 | Case control | Comparison of manoeuvres | 3 | 7 | Computed tomography | Biomechanical/cavaderic |
|  | Thailand | 2009 | 20 | Test-retest | Comparison of measurement methods | 2 | 20 | Universal goniometer  Gravity goniometer | 2 raters |
|  | Israel | 2010 | 21 | Test-retest | Comparison of measurement methods | 2 | 30 | Digital inclinometer | Seated only in one condition |
|  | Canada | 2006 | 22 | Test-retest | Comparison of measurement methods | 2 | 55 | Inclinometer  Optoelectronic | Seated only |
|  | Canada | 2010 | 23 | Case control | Comparison of measurement methods | 2 | 20 | Inclinometer  Electromagnetic measurement | Laboratory setting |
|  | UK | 2010 | 24 | Case control | Comparison of measurement methods | 4 | 100 | Visual estimation  Tape measure  Universal goniometer  Inclinometer | Laboratory setting |
|  | UK | 2010 | 25 | Systematic Review | Cervical motion assessment methodology | N/A | 56 | Reliability studies  Validity studies | Ambiguous study quality for reviewed papers |
|  | UK | 2010 | 26 | Test-retest | Comparison of conditions | 2 | 26 | Inclinometer | Physiotherapy focus |
|  | UK | 2011 | 27 | Case control | Collar comparison | 3 | 50 | Goniometer | Seated only |
|  | USA | 2007 | 28 | Case control | Device comparison | 7 | 45 | Digital Inclinometer | Single rater |
|  | Netherlands | 2008 | 29 | Case control | Comparison of measurement methods | 2 | 50 | Digital Inclinometer  Electromagnetic measurement | No simultaneous assessment |
|  | Greece | 2009 | 30 | Case control | Comparison of measurement methods | 3 | 10 | Digital Inclinometer  Electromagnetic measurement | Seated only |
|  | Australia | 2005 | 31 | Repeated measures | Comparison of measurement methods | 2 | 4 | Digital Inclinometer  Electromagnetic measurement | Seated only |
|  | USA | 2009 | 32 | Repeated measures | Device comparison | 2 | 8 | Electromagnetic measurement | Biomechanical/cavaderic |
|  | USA | 2009 | 33 | Repeated measures | Comparison of manoeuvres | 2 | 4 | Electromagnetic measurement | Biomechanical/cavaderic |
|  | USA | 2011 | 34 | Repeated measures | Comparison of manoeuvres | 2 | 5 | Electromagnetic measurement | Biomechanical/cavaderic |
|  | USA | 2010 | 35 | Test-retest | Device comparison | 2 | 1 | Radiography | Biomechanical |
|  | USA | 2010 | 36 | Repeated measures | Comparison of manoeuvres | 2 | 3 | Electromagnetic measurement | Biomechanical/cavaderic |
|  | Australia | 2003 | 37 | Test-retest | Intra-testing reliability | 2 | 15 | Electromagnetic measurement | Seated only |
|  | USA | 2004 | 38 | Repeated measures | Collar comparison | 3 | 5 | Electromagnetic measurement | Biomechanical/cavaderic |
|  | Australia | 2007 | 39 | Case control | Comparison of measurement methods (2) |  | 10 | Electromagnetic measurement | Seated only |
